# Screening and supporting through schools: educational experiences and needs of adolescents living with HIV in a South African cohort

**DOI:** 10.1186/s12889-019-6580-0

**Published:** 2019-03-06

**Authors:** Elona Toska, Lucie Cluver, Mark Orkin, Anurita Bains, Lorraine Sherr, McKenzie Berezin, Laurie Gulaid

**Affiliations:** 1 AIDS and Society Research Unit, University of Cape Town, Cape Town, South Africa; 2 Department of Sociology, University of Cape Town, Cape Town, South Africa; 3 Department of Social Policy and Intervention, University of Oxford, Oxford, UK; 4 Department of Psychiatry and Mental Health, University of Cape Town, Cape Town, South Africa; 5 MRC-NRF Developmental Pathways to Health Research Unit, School of Clinical Medicine, University of Witwatersrand, Johannesburg, South Africa; 6 UNICEF Eastern and Southern Africa, Nairobi, Kenya; 7 UNICEF Eastern and Southern Africa, Johannesburg, South Africa; 8 Research Department of Global Health, University College London, London, UK; 9 Department of Applied Psychology, New York University, New York, NY USA

**Keywords:** HIV/AIDS, Adolescents, Education, Health, Path analyses, South Africa

## Abstract

**Background:**

Many adolescents living with HIV remain disconnected from care, especially in high-prevalence settings. Slow progressors–adolescents infected perinatally who survive without access to lifesaving treatment–remain unidentified and disconnected from heath systems, especially in high-prevalence settings. This study examines differences in educational outcomes for ALHIV, in order to i) identify educational markers for targeting HIV testing, counselling and linkages to care, and ii) to identify essential foci of educational support for ALHIV.

**Methods:**

Quantitative interviews with *N* = 1063 adolescents living with HIV and *N* = 456 HIV-free community control adolescents (10–19 year olds) included educational experiences (enrolment, fee-free school, school feeding schemes, absenteeism, achievement), physical health, cognitive difficulties, mental health challenges (depression, stigma, and trauma), missing school to attend clinic appointments, and socio-demographic characteristics. Voluntary informed consent was obtained from adolescents and caregivers (when adolescent < 18 years old). Analyses included multivariate logistic regressions, controlling for socio-demographic covariates, and structural equation modelling using STATA15.

**Results:**

ALHIV reported accessing educational services (enrolment, free schools, school feeding schemes) at the same rates as other adolescents (94, 30, and 92% respectively), suggesting that school is a valuable site for identification. Living with HIV was associated with poorer attendance (aOR = 1.7 95%CI1.1–2.6) and educational delay (aOR1.7 95%CI1.3–2.2). Adolescents who reported educational delay were more likely to be older, male, chronically sick and report more cognitive difficulties. A path model with excellent model fit (RMSEA = 0.027, CFI 0.984, TLI 0.952) indicated that living with HIV was associated with a series of poor physical, mental and cognitive health issues which led to worse educational experiences.

**Conclusion:**

Schools may provide an important opportunity to identify unreached adolescents living with HIV and link them into care, focusing on adolescents with poor attendance, frequent sickness, low mood and slow learning. Key school-based markers for identifying unreached adolescents living with HIV may be low attendance, frequent sickness, low mood and slow learning. Improved linkages to care for adolescents living with HIV, in particular educational support services, are necessary to support scholastic achievement and long-term well-being, by helping them to cope with physical, emotional and cognitive difficulties.

## Background

Early HIV testing and treatment is crucial to the survival and long-term well-being of adolescents living with HIV [1]. Early detection and initiation in care is particularly relevant in Sub-Saharan Africa, where more than 80% of adolescents living with HIV reside [2], and where AIDS-related illness is the main cause of mortality among 10–19 year-olds [3]. Improved rollout of antiretroviral therapy (ART) in the region has resulted in growing coverage among adolescents living with HIV, yet less than half of adolescents under 15 reported being on ART [4], with rates among older adolescents estimated to be even lower [5], although data is only available from a small number of countries. Moreover, less than a third of adolescents in Eastern and Southern African had ever been tested for HIV and knew the results of their most recent HIV test [6]. To end AIDS as a public health threat, it is important that all adolescents living with HIV are reached for testing, treatment, care and support.

However, many adolescents, including those who have lived with HIV from birth but not yet accessed treatment and those newly-infected, remain unidentified and disconnected from health systems, especially in high-prevalence settings. Modelling of data from the region suggests that ‘slow progressors’ – young people able to survive until late adolescence, despite lack of ART initiation – account for a third of adolescents living with HIV [7]. These slow progressors may have poorer health due to delayed access to HIV testing, treatment and care. Recent estimates suggest that less than half of young people in Malawi, South Africa, Zambia and Zimbabwe know their HIV status [8, 9]. There is therefore, a significant gap in linking newly-infected adolescents to testing, treatment and care. Moreover, once tested for HIV, adolescents experience poor linkages to care, including delayed ART initiation and return HIV testing for those at high-risk of infection [5, 9–12].

School-based HIV testing is one option for detecting undiagnosed HIV cases, and has been piloted in South Africa and other countries [13–17]. However, this approach may not be feasible at a population level, due to the considerable material, medical and human resources required [18, 19]. Additionally, evaluations suggest that large-scale school-based testing programmes in limited resource settings may not lead to strong linkages to care for those who test HIV-positive [18]. Cost-effective and low-stigma strategies to identify and link undiagnosed HIV-infected adolescents to care are needed.

There is some evidence that symptomatic and behavioural screening may have potential to identify high-risk adolescents for testing and linkage to care. Systematic reviews [20–22] have documented poorer cognitive and developmental experiences among children and adolescents living with HIV in limited-resource settings. Research in the United States suggests that tailored screening of school-related experiences such as absenteeism and poor educational attainment may be effective in identifying mental health issues [23, 24]. However, no known studies have examined school experiences among adolescents living with HIV in Sub-Saharan Africa, nor how these may have the potential to improve linkages to care for adolescents living with HIV. In addition, schools in Sub-Saharan Africa report substantial barriers to providing support to adolescents living with HIV. Many adolescents and their families do not wish to disclose HIV status at school for fears of non-confidentiality and stigma. Although systematic reviews suggest evidence of cognitive challenges for children with HIV [22], there is little evidence to identify the specific educational needs of adolescents living with HIV.

Evidence on the effect of HIV on the educational outcomes of adolescents living with HIV in sub-Saharan Africa is limited. Studies which are mostly smaller samples or case control studies report inconsistent findings. A study of adolescents living with HIV and HIV-related orphans in Zimbabwe found that orphanhood drove poorer educational outcomes, but this was most likely due to the very small number of adolescents living with HIV in the study [25]. Most studies on educational outcomes and experiences of adolescents living with HIV are qualitative or quantitative studies with small sample sizes of mostly younger adolescents living with HIV. A recent meta-analysis identified three cognitive domains that HIV may negatively affect: working memory, processing speed and executive function [20]. This analysis confirmed the findings of a 2014 systematic review which found detrimental effects on cognitive development of HIV among children and adolescents, which could be attenuated by early access to ART [21]. Finding and supporting all adolescents living with HIV to thrive at home and school is critical to their long-term well-being. We need a better understanding of the pathways to educational experiences among adolescents living with HIV, and how these educational experiences can support the identification of adolescents who need treatment, care and support.

This study examines differences in educational experiences in an HIV-status assorted community-traced sample of adolescents in South Africa, in order to i) propose educational markers for prioritizing HIV testing, counselling and linkages to care, and ii) identify essential foci of educational support for adolescents living with HIV.

## Methods

This study interviewed 1519 adolescents comprising *N* = 1063 adolescents living with HIV and *N* = 456 un-infected community control adolescents (aged 10–19) in South Africa from 2014 to 2015. The study was designed in collaboration with the South African national Departments of Health, Social Development and Basic Education, UNICEF, and UNAIDS. Ethical approvals were obtained from Universities of Oxford (SSD/CUREC2/12–21), Cape Town (CSSR 2013/14), provincial department of Basic Education (04/04/2014) and Health (29/08/2013), and participating facilities.

### Sample

The study population included all adolescents ever initiated onto ART in all government-run health facilities (*n* = 53) in a municipal district of South Africa’s Eastern Cape Province. From 1176 patient files found, 90.3% (*N* = 1063) – were interviewed (4.1% refusals, 3.7% unreachable, 0.9% excluded due to severe cognitive delays). Included adolescents did not differ by age, gender and rural residence from those not reached [26]. Adolescents were interviewed in the communities where they lived (over 180 villages, wards, and neighbourhoods). Participating adolescents attended one of 415 schools in the district.

To prevent potential stigmatisation of adolescents living with HIV, cohabiting or neighbouring adolescents who met age criteria were also interviewed (*n* = 456, 94.5% interviewed, 0.2% refusals, 5.3% were not traceable due to missing or incorrect contact details). HIV-status was categorised through a sequential confirmation process. First, HIV-positive status was verified through hand-searching of patient files and Tier. Net records in all health facilities where adolescents reported receiving care (*n* = 70 facilities), including two provincial hospitals, two regional hospitals, and five community health centres, responsible for initiating most patients on ART in the area [27]. If an adolescent ART file or record of having tested HIV-positive was located, participants were recoded as living with HIV. Second, adolescents without medical records of HIV status were screened using an evidence-based health symptoms and health history tool, developed to pick up suspected cases of HIV even among adolescents living with HIV who may be ‘slow progressors’ [28, 29]. Adolescents who did not meet the suspected HIV symptoms nor health history and had never tested HIV-positive based on medical records or self-reports, were interviewed and assigned HIV-negative status. The final sample included in this study included *N* = 1063 adolescents living with HIV and *N* = 456 HIV-uninfected peers.

### Procedures

Interviews were conducted by a local team trained in sensitive and ethical research with HIV-affected children and adolescents. Questionnaires were administered on tablets to minimise missing data and improve data quality [30, 31]. Informed voluntary written consent was obtained from both adolescents and caregivers (when adolescents were < 18 years old). Adolescents could withdraw from the research at any time. Linkages to healthcare and psychosocial services were provided for adolescents reporting any severe risk of harm, including defaulting from ART, symptomatic untreated TB, mental health issues such as suicidality or severe depression, and need to access special schools due to cognitive issues (*N* = 94, 6.2% of the full sample, *N* = 68, 6.4% among adolescents living with HIV and *n* = 26 (5.7%) of the comparison group. Referrals were discussed with a research team led by a social worker. All adolescents received a participation certificate and small pack of snacks and toiletries, regardless of whether they consented or completed their interviews.

### Scales and measures

Validated scales and measures were used where available, with adaptations made in consultation with adolescents, local health providers and other stakeholders. All measures were translated and back-translated, with consultation with rural and urban youth (*n* = 12) to ensure conceptual and contextual relevance between the English and the local vernacular (both urban and rural Xhosa). Final data collection tools were pre-piloted with *N* = 25 adolescents living with HIV from rural and urban study areas.

#### Educational experiences

*School enrolment*, measured as self-reported attendance in school. *Access to a fee-free school* was measured as no fee schools or adolescents reporting a school-fee waiver [32–34]. *Access to a school feeding scheme* was measured through adolescent reporting receiving at least one free meal at school (breakfast, lunch or snack), using an item developed through consultations with Department of Basic Education. *Absenteeism* was dichotomised as missing school for > 2 weeks in the previous term [35]. *Educational delay* was dichotomised using self-reported educational delay by ≥1 year behind school grade [35].

#### Physical health

*Chronic illness* used adolescent report of symptomatic pulmonary TB, frequent ear infections, difficulties breathing, diarrhoea or nausea: all health issues shown to negatively affect school attendance and performance [36–38]. *Missing school frequently to go to the clinic* in the past year was documented through an item adapted from the PREPARE trial [39].

#### Cognitive difficulties

Cognitive difficulties were dichotomised as adolescent self-reported difficulties remembering to take their medicine – adapted from the WHO International Classification of Disability [40], caregiver-reported difficulty to concentrate at school and home, or attending a special school for children with cognitive disabilities (reported by either participant or caregiver). Answering ‘yes’ to either of these three types of difficulty was coded as reporting cognitive difficulties.

#### Psychosocial well-being measures

*Mental health challenges* were measured as adolescent reporting at least one of three mental health states – depression, Post-Traumatic Stress Disorder (PTSD), or internalised stigma. Depression was measured as reporting above-median scores in the 10-item Child Development Inventory (past two weeks, Macdonald’s ω = 0.61), used extensively with children and adolescents in South Africa [41]. Past-month PTSD was measured with an abbreviated version of the Child PTSD Checklist [42], validated for use among adolescents in South Africa [43]. Internalised stigma used a scale adapted and validated with the study sample (α = 0.75) [26]. In addition, s*chool-related externalising* problems were measured by combining 2 school-related items from the Child Behaviour Checklist (CBCL) [44, 45], used in South Africa [46, 47] with 2 school-specific behaviour items from the Strengths and Difficulties Questionnaire (SDQ), validated in Xhosa speaking populations [48].

#### Socio-demographic variables

Socio-demographic variables included age, gender, rural/urban residence, informal/formal housing, and orphanhood (maternal and paternal), measured through items used in similar surveys with adolescents in South Africa [46, 49]. Poverty was measured as lacking at least one of eight top socially-perceived necessities for children and adolescents, confirmed by over 80% of the South African population in a nationally-representative survey [50].

### Analyses

**Fig. 1:**
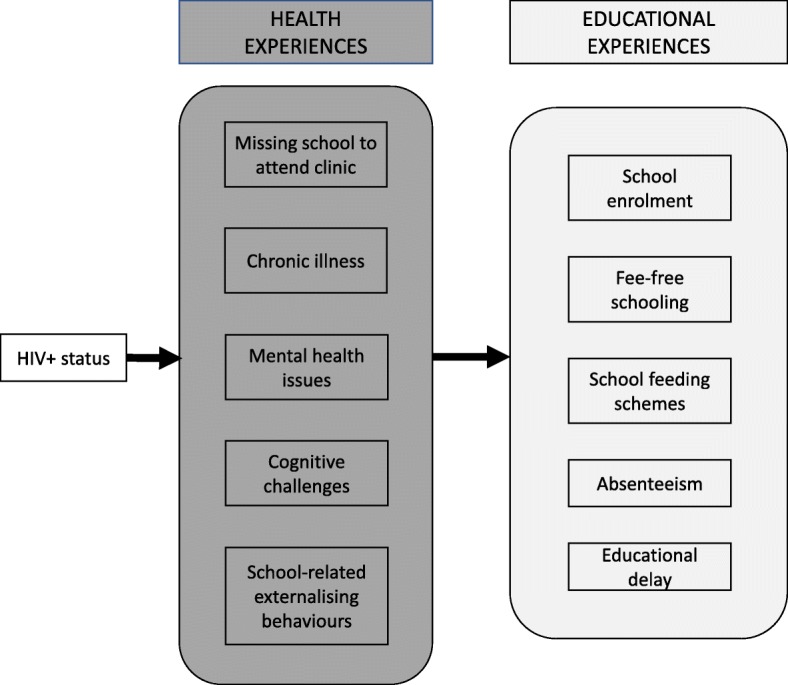
Hypothesised pathways between HIV status and educational experiences

Analyses took place in three stages using SPSS23 and STATA15:

1. Rates of socio-demographic characteristics, physical, mental and cognitive health variables, and educational experiences were computed for the full sample, followed by Chi-square tests investigating differences by HIV status.
2. Potential differences in adolescent educational experiences by HIV status were tested using multivariate regressions controlling for socio-demographic and health variables that differed by HIV-status in the first stage of analyses. Benjamini-Hochberg step-down adjustments were conducted for multiple outcome testing with *p* < 0.05 detection power, to minimize false detection rate [51].
3. All educational experiences and health variables significantly associated with HIV-positive status were included in a hypothesised exploratory path model to investigate pathways between HIV-positive status and the resulting educational experiences (Fig. 1). Non-significant pathways were eliminated sequentially, with the best fit model retained. Model fit for the tested path model was assessed as good if RMSEA (< 0.05), TLI (> 0.95), CFI (> 0.95), and χ^2^/df < 5.

## Results

### Sample characteristics

In the full sample (Table 1), adolescent mean age was 14.1 years, (SD = 2.9), were female (57%), and urban or peri-urban dwellers (77%). One in five participants lived in informal housing, with two thirds (66%) lacking at least one of eight basic necessities such as food or soap. Cognitive difficulties were prevalent in 35.6%, and mental health challenges were experienced by nearly half (46.7%). Over half were orphaned (52%): 37% maternally bereaved and 28% paternally bereaved. School enrolment was high (94% overall). Adolescents living with HIV reported higher rates of health issues (*p* ≤ 0.001), but rates of school-related externalising issues did not differ by HIV status (*p* = 0.820). School enrolment and access to fee-free schools were not different by HIV status (*p* = 0.564 and *p* = 0.314, respectively), while adolescents living with HIV reported higher rates of accessing school feeding schemes, absenteeism, and educational delay (*p* < 0.05). Adolescents living with HIV were significantly more likely to report cognitive difficulties (40.2% versus 25.4%) and mental health challenges (49.7% versus 40.2%) both significant at *p* < .001.

**Table 1:** Sample characteristics by HIV status (*N* = 1519)

Group	Variable	Total N (%) or mean (SD)	Adolescent living with HIV N (%) or mean (SD)	Community controls N (%) or mean (SD)	Chi2/ F *p*-value
Socio-demographic characteristics	**Age (years)**	**14.1 (2.9)**	**13.8 (2.8)**	**14.6 (3.0)**	**<.001**
	**Gender (female)**	**863 (56.8%)**	**576 (54.8%)**	**287 (61.2%)**	**.021**
	Rural residence	353 (23.3%)	237 (22.7%)	116 (24.7%)	.387
	Informal housing	271 (17.9%)	198 (18.9%)	73 (15.6%)	.120
	Poverty	1007 (66.3%)	708 (67.4%)	299 (63.9%)	.178
	Migration	1.96 (1.3)	1.96 (1.3)	1.99 (1.3)	.672
	**Maternal orphan**	**562 (37.0%)**	**460 (43.8%)**	**102 (21.7%)**	**<.001**
	**Paternal orphan**	**424 (27.9%)**	**318 (30.3%)**	**106 (22.6%)**	**.002**
Health experiences	**Chronic illness**	**288 (19.0%)**	**246 (23.4%)**	**42 (9.0%)**	**<.001**
	**Missing school to attend clinic**	**210 (13.8%)**	**195 (18.6%)**	**15 (3.2%)**	**<.001**
	**Cognitive difficulties**	**541 (35.6%)**	**422 (40.2%)**	**119 (25.4%)**	**<.001**
	**Mental health challenges**	**710 (46.7%)**	**522 (49.7%)**	**188 (40.2%)**	**.001**
	Externalising issues	814 (53.6%)	561 (53.4%)	253 (54.1%)	.820
Educational experiences	School enrolment	1429 (94.1%)	986 (93.9%)	443 (94.7%)	.564
	Fee-free school	450 (29.6%)	303 (28.9%)	147 (31.4%)	.314
	School feeding schemes	1396 (91.9%)	976 (93.0%)	420 (89.7%)	.034
	Absenteeism	180 (11.8%)	136 (13.0%)	44 (9.4%)	.048
	Educational delay	837 (55.1%)	605 (57.6%)	232 (49.6%)	.004

Note: *p* < 0.05 are bolded

### Effect of HIV status on health and educational experiences

In multivariate regression of educational experiences on HIV status, controlling for socio-demographic and health variables (Table 2), adolescents living with HIV were more likely to report past-term absenteeism (aOR = 1.69 95%CI 1.08–2.64 *p* = 0.021) and educational delay – being at least one grade behind (aOR = 1.65 95%CI 1.25–2.17 *p* < 0.001). HIV status was not associated with accessing school feeding schemes (*p* = 0.265) and was thus not carried forward into the path modelling.

**Table 2:** Socio-demographic characteristics associated with educational experiences by HIV status (*N* = 1519)

Characteristic	N(%) Mean (SD)	School feeding scheme OR {95%CI) p*	Absenteeism OR {95%CI) p*	Educational delay OR {95%CI) p*
Age (years)	14.1 (2.9)	**0.882 (0.825–0.943) < 0.001**	**1.461 (1.363–1.566) < 0.001**	**1.333 (1.276–1.392) < 0.001**
Gender (female)	863 (56.8%)	**0.61 (0.401–0.928) 0.021**	1.392 (0.958–2.021) 0.082	**0.638 (0.509–0.801) < 0.001**
Maternal orphan	562 (37.0%)	**1.658 (1.072–2.562) 0.023**	**0.545 (0.375–0.79) 0.001**	1.066 (0.843–1.349) 0.592
Paternal Orphan	424 (27.9%)	1.333 (0.851–2.089) 0.209	0.854 (0.587–1.242) 0.408	1.228 (0.954–1.581) 0.11
HIV-positive status	1050 (69.1%)	1.286 (0.823–2.007) 0.269	**1.69 (1.082–2.641) 0.021**	**1.652 (1.251–2.183) < 0.001**
Chronic Illness	288 (19.0%)	1.427 (0.832–2.447) 0.197	**1.694 (1.138–2.522) 0.009**	**1.641 (1.219–2.209) 0.001**
Cognitive difficulties	541 (35.6%)	1.204 (0.62–2.338) 0.583	**2.374 (1.481–3.805) < 0.001**	1.304 (0.938–1.813) 0.114
Mental health challenges	710 (46.7%)	0.814 (0.546–1.213) 0.311	**1.676 (1.181–2.379) 0.004**	**1.332 (1.05–1.689) 0.018**
Missing school most of the time	210 (13.8%)	0.636 (0.389–1.041) 0.072	1.047 (0.663–1.654) 0.843	0.824 (0.629–1.08) 0.161

* *p*-values adjusted using the Benjamini-Hochberg stepdown approach for multiple testing

Note: *p* < 0.05 are bolded

School absenteeism was also associated with older age (aOR = 1.46 95%CI 1.36–1.57 *p* < 0.001), maternal orphanhood (aOR = 0.55 95%CI 0.38–0.79 *p* = 0.001), being chronically sick (aOR = 1.69 95%CI 1.14–2.52 *p* = 0.009), missing school often to attend clinics (aOR = 2.37 95%CI 1.48–3.81), and greater cognitive difficulties (aOR = 1.68 95C%CI 1.18–2.38 *p* = 0.004). Educational delay was also significantly associated with older age (aOR = 1.33 95%CI 1.28–1.39 *p* < 0.001), being male (aOR = 1.57 95%CI 1.25–1.96 *p* < 0.001), being chronically sick (aOR = 1.64 95%CI 1.22–2.21 *p* = 0.001), and greater mental health difficulties (aOR = 1.33 95C%CI 1.05–1.69 *p* = 0.018).

### Pathways from adolescent HIV status to educational experiences

All health variables associated with HIV-positive status, plus school absenteeism and educational delay, were entered in a path model. Living with HIV was hypothesised to directly affect *experiences:* absenteeism, educational delay, chronic illness, cognitive difficulties, mental health challenges, and needing to miss school to attend clinics. Unidirectional relationships were hypothesised of HIV-status leading to chronic illness, cognitive difficulties and mental health challenges. Bidirectional relationships were hypothesised between cognitive difficulties, chronic illness, and mental health challenges.

**Fig. 2:**
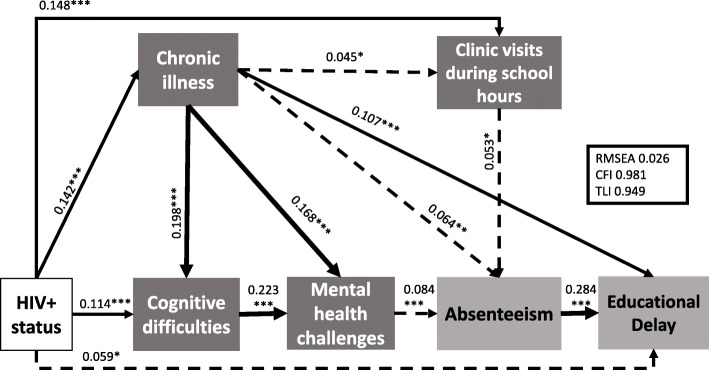
Cascade from HIV Status to educational outcomes

The model identified both direct and indirect pathways. Adolescents living with HIV experienced higher levels of cognitive difficulties (β = 0.114, *p* < 0.001), which in turn were associated with worse mental health (β = 0.223, *p* < 0.001), higher rates of absenteeism (β = 0.084, *p* < 0.001) and educational delay (β = 0.284, *p* < 0.001). In a separate branch of the model, adolescents living with HIV experienced worse physical health issues (β = 0.142, *p* < 0.001), which exacerbated cognitive difficulties (β = 0.198, *p* < 0.001), mental health (β = 0.168, *p* < 0.001), absenteeism (β = 0.064, *p* < 0.001) and educational delay (β = 0.107, *p* < 0.001). Having a higher disease burden resulted in more frequent need to miss school to attend clinics (β = 0.045, *p* < 0.045), and higher absenteeism (β = 0.053, *p* < 0.5). Living with HIV and experiencing chronic illness remained directly associated with educational delay (β = 0.142, *p* < 0.001 and β = 0.059, *p* < 0.05, respectively). HIV-positive status was also directly associated with missing school to attend clinic appointments (β = 0.148, p < 0.001). The final model (Fig. 2) had good fit for all pre-selected criteria (RMSEA = 0.026, CFI = 0.981 and TLI = 0.949, χ^2^(df) = 16.10 [8], χ^2^/df = 2.01), after all non-significant pathways were removed. All constructs included in the model were observed variables. Values indicate unstandardised β weights. All non-significant pathways were removed. Full lines indicate significant pathways at the *p* < 0.001 (***), *p* < 0.01 (**), and *p* < 0.05 (*) level.

## Discussion

This study aimed to identify 1) potential school-based markers to target HIV testing, counselling and treatment and 2) areas in which adolescents living with HIV may benefit from support to improve their educational experiences. First, we documented the educational experiences of adolescents living with HIV. Notably, adolescents living with HIV reported similar school enrolment, access to fee-free schools, and school feeding schemes to their uninfected peers, controlling for socio-demographic characteristics and health experiences. These results substantiate South Africa’s significant gains in rolling out educational services to all adolescents, including the most vulnerable. They also suggest that school-based identification of adolescents at risk of undiagnosed HIV-infection may be of value. However, with 7% of adolescents living with HIV not attending school, community-based outreach remains essential to reach those who are potentially most vulnerable.

Living with HIV was strongly associated with absenteeism and educational delay, controlling for potential co-factors. Adolescents living with HIV reported more chronic illness, cognitive difficulties and mental health challenges of depression and stigma. These health problems may be helpful markers to support education services in linking adolescents to HIV testing services where needed. An estimated third of adolescents living with HIV in sub-Saharan Africa survive to a median age of 16 without access to ART [7] although they experience poor health [52]. Rates of diagnosis for those infected during adolescence (except for ante-natal testing) remain low. Emerging evidence from a two-country HIV testing and counselling trial (Pop-ART) suggests that using community-based HIV testing may not be the most cost-effective way to identify undiagnosed adolescents [53]. Our findings suggest that schools may provide low-cost opportunities for identifying highly vulnerable adolescents who may benefit from targeted testing and linkages to care. Living with HIV was strongly associated with absenteeism and educational delay, controlling for potential co-factors. Adolescents living with HIV reported more chronic illness, cognitive difficulties and mental health challenges of depression and stigma. These health problems may be helpful markers to support education services in linking adolescents to HIV testing services where needed. An estimated third of adolescents living with HIV in sub-Saharan Africa survive to a median age of 16 without access to ART [7] although they experience poor health [52]. Rates of diagnosis for those infected during adolescence (except for ante-natal testing) remain low. Emerging evidence from a two-country HIV testing and counselling trial (Pop-ART) suggests that using community-based HIV testing may not be the most cost-effective way to identify undiagnosed uninitiated adolescents [53]. Our findings suggest that schools may provide low-cost opportunities for identifying highly vulnerable adolescents who may benefit from targeted testing and linkages to care.

The study also investigated ways that schools could support adolescents living with HIV. We used multivariate models, followed by an exploratory structural pathway between HIV-status, health issues and educational experiences. These showed that cognitive difficulties and chronic illness, both experienced in higher levels by adolescents living with HIV, set off cascades of effects resulting in poorer educational experiences: more absenteeism and educational delay. Our findings suggest needs for specialised support in schools can be identified without educational services focusing on or knowing an adolescent’s HIV status. School-based educational support – for example the work of Learning Support Agents in South Africa – could be offered to adolescents who have worse attendance, frequent sickness, low mood, and difficulties concentrating at school and learning – thus also reaching those who are HIV-infected. Secondly, findings suggest that pathways to negative educational experiences could be interrupted through interventions that improve overall mental, physical, and emotional health. Additional support for adolescents with learning difficulties and frequent sickness may facilitate remaining in school.

Findings also suggest that adolescents struggle to maintain retention in HIV-care and in school. Frequent clinic appointments were associated with reduced school attendance and educational delay, even when considering indirect pathways through poorer health. Recent innovations in paediatric and adolescent HIV-care suggest approaches that may reduce waiting times and transport time for adolescents, including fast-track clinic queues for school learners (used by some clinics in the study area), adolescent-specific opening hours, and public transport subsidies [54].

This study has several limitations. First, it uses cross-sectional data, and thus limits our ability to determine causal relationships. However, we included several bi-directional relationships in the path model tested, which ruled out some of the possible reverse causalities in the significant relationships. Second, although HIV-positive status was verified through Tier. Net records and patient files in > 70 facilities in the study area, we used self-reported data for other health and educational measures. Linking adolescents’ health data with official educational records could help confirm educational achievement experiences, but such data was not available for schools in the research catchment. Third, this study focused on physical, mental and cognitive health markers of HIV-infection and educational needs. Other potential family-based markers – such as orphanhood and family poverty – may be important indicators, but were not included as consultations suggested that school staff may be less aware of these for adolescent students in large classes and in communities where both poverty and orphanhood are highly prevalent, such as those where this study was conducted. Moreover, neither poverty nor orphanhood were associated with educational delay in multivariate analyses. Fourth, participants with severe cognitive delays (0.9%) were unable to respond to the surveys. Study findings likely underestimate the burden and effect of cognitive issues on related outcomes among the general adolescents living with HIV population.

## Conclusion

This is the first known study to identify differences in educational experiences for adolescents living with HIV in resource-limited settings. Findings suggest potential for identifying unreached adolescents living with HIV through school-based markers: of low attendance, repeated grades, frequent sickness, low mood and learning difficulties. Findings also suggest that adolescents living with HIV experience substantial educational difficulties that could benefit from targeted and sensitive support. By supporting adolescents with physical, emotional and cognitive difficulties with educational assistance, schools can reach those affected without requiring unwanted disclosure of HIV-status. If we are to give adolescents living with HIV the best opportunities for successful adulthood, it is essential that educational systems are strengthened and capacitated to support this group.

## Abbreviations

AIDS: Acquired Immunodeficiency Syndrome
ART: Antiretroviral therapy
HIV: Human Immunodeficiency Virus
